# Emerging roles of infiltrating granulocytes and monocytes in homeostasis

**DOI:** 10.1007/s00018-020-03509-8

**Published:** 2020-04-04

**Authors:** Linda Groeneweg, Andres Hidalgo, Noelia A-Gonzalez

**Affiliations:** 1 Institute of Immunology, University of Münster, Münster, Germany; 2 Cells-in-Motion Interfaculty Center, University of Münster, Münster, Germany; 3 Department of Cell and Developmental Biology, Centro Nacional de Investigaciones Cardiovasculares (CNIC) Carlos III, Madrid, Spain; 4 Institute for Cardiovascular Prevention (IPEK), Ludwig Maximilians University, Munich, Germany

**Keywords:** Myeloid cells, Tissue infiltration, Neutrophils, Tissue microenvironment, Cell migration

## Abstract

The infiltration of naïve tissues by myeloid cells has been long related to their clearance and the physiological cell turnover, however, increasing evidence shows that they can additionally fulfill specific, non-immune functions in different tissues. There is also growing evidence to support that infiltrated granulocytes and monocytes respond to different environments by modulating gene expression and cytokine production, which in turn contribute to the normal function of the host tissue. This review will address the roles of immigrated myeloid cells in different tissues and their crosstalk with the host tissue environments.

## Introduction

The generation of an effective immune response typically starts with the infiltration of damaged tissues by neutrophils and monocytes. These cells migrate to the inflamed tissues following cytokine and chemokine gradients that are mainly produced by other sentinel, tissue-resident, myeloid cells [1]. Nonetheless, the infiltration of naïve tissues by myeloid cells as an active mechanism of organ homeostasis has recently become apparent [2, 3]. Historically, the migration of myeloid cells into naïve tissues has been attributed to the necessity of these cells to be cleared, particularly in the case of neutrophils, or to replace other cell types, for example monocytes that occupy niches of eliminated macrophages or dendritic cells (DC), all of which is part of the physiological cell turnover. However, it has only recently been acknowledged that myeloid cells also migrate to naïve tissues to contribute to normal tissue function. Several studies have attributed a regulatory role to myeloid cells in homeostasis, for example by promoting immune tolerance via the interaction with other cell types, such as the generation of regulatory T cells in the intestine through macrophage-derived IL-10 [4], or antibody production by B cells in the marginal zone of the spleen by infiltrating neutrophils [5]. Leukocytes follow circadian rhythmicity in their homeostatic migration, which is partially regulated by the host tissue in response to organ-specific environmental cues [6]. Nevertheless, different organs can also share particular microenvironments, such as secondary lymphoid organs and the digestive tract, in which lymphoid follicles are present. Indeed, several studies show that preferred locations of infiltrating neutrophils are the marginal zone of the spleen, which surrounds the B/T cell follicles [5], and around the isolated patches in the large intestine [2]. Neutrophils in the marginal zone of the spleen produce cytokines that promote somatic hypermutation and immunoglobulin A (IgA) production by marginal zone B cells [5]. The similar location of neutrophils in the intestines surrounding lymphoid follicles suggests a similar crosstalk with intestinal B cells regulating IgA production. Hence, it will be important to define whether infiltrating myeloid cells generally regulate B cell functions in other organs containing lymphoid clusters, a notion that would in turn set a paradigm for the functional adaptions of infiltrating myeloid cells to specific microenvironments. In this review we will provide an overview of current knowledge on the functions of immigrated myeloid cell, with a special focus on granulocytes, to naïve tissues beyond the immune responses, and will discuss the contribution of circulating myeloid cells to the identity of the organs that they infiltrate.

## Regulatory functions of neutrophils through clearance

Neutrophils originate in the bone marrow (BM) from hematopoietic stem cells that give rise to granulocytic progenitors, which in turn proliferate and differentiate to fully mature neutrophils. Around 90% of the neutrophil pool remains in the BM 4–6 days post-differentiation [7], available for their rapid release and mobilization on demand, such as during inflammation. Several signaling pathways and cell types are implicated in the release, circulation and migration of neutrophils. The interaction between CXCL12 and CXCR4 mediates the retention and homing of neutrophils within the BM while their mobilization into the circulation is triggered by G-CSF, which induces reductions in CXCR4 signaling in the marrow [8, 9]. In the absence of inflammatory signals, neutrophil lifespan is estimated in about 12 h in mice, with controversial studies suggesting up to 5.4 days in humans [10]. Interestingly, their lifecycle in blood follows circadian rhythms [11–13]. During their time in the circulation there is a dynamic phenotypic switch, with increased CXCR4 and decreased CD62L expression, through a process referred to as neutrophil ageing [14, 15]. Neutrophils at the end of their life cycle, referred to here as aged neutrophils, show additional alterations in the expression of receptors associated with activation, adhesion and cell death, such as CD11b, CD49d and CD47 [13, 16–18]. If no tissue damage or inflammatory stimuli is detected by the neutrophils during their time in circulation, once they reach the end of life cycle, they infiltrate the bone marrow, spleen and liver, among other organs [19], where they are cleared by tissue-resident macrophages. When aged neutrophils are cleared from the circulation in the bone marrow, they are phagocytosed by tissue-resident macrophages, which generate homeostatic signals that modulate the haematopoietic niche. Engulfment of neutrophils by tissue-resident macrophages results in reduction of CXCL12 production by stromal cells, followed by moderate egress of haematopoietic progenitor cells into the bloodstream [14]. Beyond the bone marrow, recent studies from our lab show that the homeostatic migration of neutrophils to the large intestine also regulates this axis, as intestinal macrophages reduce the expression of IL-23 upon phagocytosis of neutrophils [2], a process controlling granulopoiesis by promoting the expression of IL-17 by lymphoid cells, which in turn regulates G-CSF production [20]. Using surgical approaches with reporter and genetically modified mice, we further demonstrated that besides the classical neutrophil clearance sites, bone marrow, spleen and liver [21, 22], extra medullary tissues such as the skin, lung or adipose tissue are infiltrated by neutrophils in the steady-state. Importantly, the immigrated neutrophils that are engulfed by tissue-resident macrophages appear to critically regulate the transcriptional profile of the engulfing macrophage, thereby supporting immune homeostasis in tissues [2, 23]. We and others have been particularly interested in the phagocytic pathways that tissue-resident macrophages display during this homeostatic clearance of neutrophils in diverse tissues. This is, however, a technically challenging problem to address as, for example, the cell death modality of neutrophils in homeostasis and whether a proportion of neutrophils exits the tissues after infiltration remain poorly defined. The general assumption that neutrophils die by apoptosis during their physiological clearance is still under debate, as neutrophils can undergo several cell death modalities, such as NETosis, pyroptosis or necroptosis, under different circumstances [24]. Due to the lack of markers of phagocytosed cells and the extremely efficient process of phagocytosis, the visualization and characterization of neutrophil phagocytosis in living tissues is somewhat challenging. Nevertheless, we have demonstrated that specifically tissue-resident macrophages, and no other population residents in the tissues, are in charge of the phagocytosis of circulating neutrophils [2, 13, 23]. In addition, we could observe that in tissues where macrophages showed impaired phagocytic capacity, there is accumulation of neutrophils in late phases of apoptosis, which indicates that, if neutrophils are not properly cleared by tissue-resident macrophages, they undergo apoptosis and may cause tissue damage by secondary necrosis [25].

## The lung as a reservoir of granulocytes with regulatory functions

It has long been acknowledged that in mice the lung functions as a marginal pool for neutrophils under homeostatic conditions. Neutrophils accumulate in pulmonary microvessels [26–28] during their diurnal circulation [2]. Specifically, it has been shown that neutrophils accumulate in the luminal surface of the pulmonary blood vessels in a CXCR4-dependent mechanism [29], where they can be rapidly mobilized in case of an inflammatory stimulus [30]. Interestingly, we showed that there is a time-dependent oscillation in the transcription of a defined group of genes that is specifically influenced by the diurnal accumulation of neutrophils in the lung [2]. These genes are mostly involved in inflammation, cell growth and metastasis, suggesting a role in carcinogenesis. Using a model of lung metastasis by melanoma cell injection, we observed more metastatic foci in the lung of mice injected in the morning. In addition, mice depleted of neutrophils showed no rhythmicity in the formation of metastatic foci in response to melanoma cell injection [2]. Although much remains to be learned on the mechanism of this transcriptional regulation of a tissue, these findings highlight the importance of the marginal pool of neutrophils in cancer cell homing to the lung, suggesting their contribution to secondary migration of other cells to peripheral tissues. Intriguingly, the specific cell types in the lung that are targeted by neutrophils during their homeostatic infiltration and whether they cooperate in the regulation of cell migration are processes that remain not fully understood. Moreover, whether factors produced by the endothelium drive their retention and accumulation in the marginal pool is still under debate. Nonetheless, the margination of granulocytes within the microvasculature of the lung does not appear to be exclusive of neutrophils. Basophils, previously thought to be dispensable in the absence of inflammation, have a lung-specific unique gene signature, which includes expression of Il6, Il13, Cxcl2, Tnf, Osm and Ccl4, and is distinct from that of circulating basophils under homeostatic conditions [31]. This phenotype is imprinted by the lung environment, namely by GM-CSF and IL-33, two molecules that promote basophil survival [32, 33] and further polarize alveolar macrophages to a phagocytic, non-inflammatory state, indicating prominent roles for these factors in global lung homeostasis.

## Vascular system: patrolling the body

A key step for the infiltration of most tissues by myeloid cells is their contact with the vasculature during the extravasation process. In the past decades, significant effort has been put into defining the cascade of events and the molecular cues implicated in leukocyte migration and extravasation during inflammation [1]. Endothelial cells and pericytes both play a key role in this process, not only through the expression of cytokine receptors, integrins and adhesion molecules, but also through the mechano-physical features that influence the squeezing and rapid changes of shape that leukocytes must acquire to transmigrate [34]. It is now accepted that these mechanisms of migration of myeloid cells are diverse and are dictated by the leukocyte type and by the environmental properties of each organ [6, 35]. Accordingly, it has been suggested that the intestinal endothelium, in response to monocyte transmigration, contributes to some specific functions of the immigrated cells once they enter into the tissue [4]. For example, when monocytes transmigrate to the intestinal parenchyma, they differentiate into macrophages, which specifically express high levels of the cytokine receptor CX3CR1. Its ligand, CX3CL1 is produced by the intestinal endothelium, and its recognition by CX3CR1 in the surface of intestinal macrophages enables sampling of luminal contents [4]. This suggests a role of the intestinal endothelium in the protective function of macrophages via the CX3CL1/CX3CR1 axis. Notably, monocytes have the potential to survey the endothelium in search of potentially dangerous material. In particular, CX3CR1^high^LyC6^low^, or non-classical, monocytes continuously monitor capillaries, arterioles and venules, crawling on endothelial cells in a LFA/ICAM-dependent mechanism and efficiently scavenging microparticles [36, 37]. Their patrolling behaviour in the microcirculation contributes to endothelial homeostasis [37–39] and has been suggested to prevent endothelial apoptosis in large arteries during atherosclerosis [40]. Using an elegant approach to visualize the patrolling behaviour of monocytes by intravital microscopy of unrestrained arteries during atherosclerosis, the authors showed that in the absence of non-classical monocytes, endothelial damage was aggravated [40]. Although the mechanism by which this protective role of non-classical monocytes in endothelial cells has not yet been deciphered, these data suggest that non-classical monocytes regulate vascular integrity, at least during inflammation. Whether non-classical monocytes also play a general protective role in arteries under steady-state conditions is an important issue that remains to be addressed.

During the immune response, monocytes that migrate to damaged or inflamed tissue can also differentiate into DCs, which, upon sensing foreign particles, migrate to draining lymph nodes to initiate an adaptive immune response. Interestingly, however, DC progenitors migrate into tissues also during embryonic and postnatal development. They infiltrate the skin, mucosal surfaces and almost all solid organs in the body, ultimately giving rise to immature conventional DC [41]. In the steady-state, these conventional DC migrate in a CCR7-dependent mechanism through the tissue and show a distinct MHC-II^hi^ CD11c^low^ phenotype in comparison to tissue-resident DCs [41]. They further induce the proliferation of lymphatic endothelial cells, through DC-derived VEGF, and the maturation of high endothelial venules, by activating the lymphotoxin-b receptor in endothelial cells [42–44]. This remodelling of the lymph node-specific vasculature is crucial for lymphocyte homing [45]. Nevertheless, the crosstalk of myeloid cells with the endothelium is also evident in other organs, such as the kidney, where podocytes in blood vessels regulate neutrophil recruitment via IL-6 secretion [46]. Altogether, these studies support the notion that the endothelium is a key structure, unique to each tissue that can potentially contribute to modulate certain functions of myeloid cells by enabling their transmigration.

In addition to monocytes, it will be important to explore whether the neutrophils contained in the so-called “marginated pool”, within blood vessels, also interact with the endothelial cells to regulate their functions. Interestingly, pro-angiogenic properties of neutrophils have already been shown upon tissue engraftment and have been proposed to involve a distinct subtype of neutrophils [47]. Christoffersson and collaborators showed in pancreas transplantation experiments that several subsets of neutrophils exist, one of them with pro-angiogenic properties. These neutrophils express high levels of CXCR4 and contribute to the formation of new capillaries in transplanted pancreatic islets via the release of MMP9 [48]. Whether neutrophils contribute to angiogenesis also during development or in other disease environments, such as in the tumor microenvironment, remains unknown; nonetheless, these studies open potential therapeutic approaches in diseases with a central contribution of the vascular system.

## Metabolic impact of neutrophils and eosinophils

Over the past decade, the study of immunometabolic pathways in myeloid cells has attracted much attention. Migrating myeloid cells have high rates of metabolic activity to fulfil the energy requirements needed for transmigration into and function within tissues. Paradoxically, the metabolic activity of myeloid cells has been often overlooked in classical metabolic tissues, such as the adipose tissue or the liver. Although neutrophils are present at low levels in steady-state in the adipose tissue [2], their influence on this tissue can be significant; for example, neutrophil-derived elastase can degrade Insulin receptor substrate 1 (IRS1) in adipocytes and hepatocytes, inducing insulin resistance and promoting lipogenesis and cholesterol synthesis [49]. Although adipose tissue is in an inflammatory state, these findings suggested that basal neutrophil infiltration in the liver and fat can potentially regulate multiple aspects of their metabolic functions. Similarly, it has been shown that eosinophils, which migrate into adipose tissue by an integrin-dependent process and are a crucial source of IL-4, maintain an anti-inflammatory milieu for adipose tissue macrophages [50, 51]. Notably, eosinophil-deficient mice fed with high-fat diet display an increase in total body fat and an improved response to glucose challenge, suggesting a role for eosinophils in protecting against diet-induced obesity [51]. Eosinophils also home to the gastrointestinal tract under physiological conditions, in a process driven by the eotaxin-1 gradients [52, 53]. In eotaxin-deficient mice, eosinophils were significantly reduced in the lamina propria [54] and accumulated in the blood [55]. In the intestine they affect gut-associated lymphoid tissue and microbiota composition, by diminishing Tfh cell function, Treg and DC development and increasing the number of Gram^−^ bacteria. Further, deficiency in eosinophils alters the development of Peyer’s patches, resulting in smaller patches that contain fewer cells, as well as fewer regulatory T cells and CD103^+^ DC, which are in turn involved in immune tolerance and the generation of IgA^+^ plasma cells [53]. Furthermore, mucus production is decreased in the small intestines of these mice [56], and IL-17A producing T helper cells are increased [57] upon eosinophil depletion. In Peyer’s patches they promote class-switch of B cells to IgA, and are implicated in the survival of plasma cells, as eosinophil depletion results in apoptosis of long-lived bone marrow plasma cells [58, 59].

## Female reproductive tract

In the female reproductive system, neutrophils are recruited to the vaginal lumen and protect against infections, in a process known to be temporally regulated by sex hormones during the estral cycle [60]. At the foetal-maternal interphace and in the mesometrial triangle in rats, neutrophils are co-localized with IL-10 producing cells [61], an anti-inflammatory cytokine, suggesting an immune protective impact in this tissue. Eosinophils, in contrast, play a crucial role during the postnatal development of the mammary glands [62] in a process driven by IL-5. In IL-5 deficient mice, fewer terminal end buds, less well-developed branching of the mammary ducts, and lower overall density of mammary gland structures have been reported [63], pointing out to the importance of eosinophil recruitment in mammary gland development. Using eotaxin-1 knockout mice, which show impaired homing of eosinophils to the uterus and mammary gland, reduced number of mammary ductal tree branches and terminal end buds were found [64]. Additionally, during pregnancy, eosinophil degranulation prepares the cervix for delivery [65]. Although these observations indicate a potential role of eosinophils in preparing the uterus for pregnancy, the subsequent estral cycle in adult mice is not affected by depletion of eosinophils [66]. Nevertheless, these findings suggest an important contribution of eosinophils to the female reproductive system. In addition, monocytes and monocyte-derived macrophages also contribute to the development and homeostasis of the female reproductive tract. Monocyte recruitment, which is a crucial event during embryonic development, is dependent on CSF-1/CCR2 interactions in the mouse female reproductive tract [67]. Indeed, in *Csf1*_*op*_*/Csf1*_*op*_ mice, which lack several tissue macrophage populations since early developmental stages, the ductal elongation during the mammary gland development is impaired [68]. Several studies showed that both during mammary gland morphogenesis and during lactation, monocytes and macrophages regulate the organization of the extracellular matrix, by providing MMP9, and the phagocytosis of cell debris, contributing to the development and normal functions of the tissue [62, 64, 69, 70].

## Monocytes and monocyte-derived macrophages in development

In the past decade, a profuse number of studies have been published regarding the ontogeny, heterogeneity and plasticity of tissue-resident macrophages (for extensive reviews see [71, 72]), highlighting the crucial role of macrophage development for their functions in the adult tissues. It is now well established that some tissue-resident macrophages originate from early embryonic progenitors and self-maintain during adulthood without the contribution of circulating monocytes. Yet, the contribution of infiltrating monocytes in the adult is a key feature for some macrophage populations, such as macrophages in the gut mucosa [73]. Intriguingly, it appears evident that the macrophage pool dependent on monocyte replenishment actively contributes to tissue-development. In the absence of monocyte replenishment of the gut macrophage pool, morphological abnormalities in the submucosal vasculature and loss of enteric neurons have been found [74]. In the kidney, fetal monocytes colonize the kidney anlagen and facilitate many processes during renal organogenesis, like branching morphogenesis [75]. In the embryonic kidney, macrophages restrict the early domain of nephron progenitor cells, by clearing rostral nephron progenitors. Upon kidney development initiation, they frequently interact with blood vessels and promote endothelial cross-connections, as their depletion results in CD31^+^ structures in the developing kidney [75]. Interestingly, it has been proposed that the colonization of macrophage precursors at embryonic day 9.5 is an integral part of the tissue organogenesis program [76].

## Conclusion

**Fig. 1:**
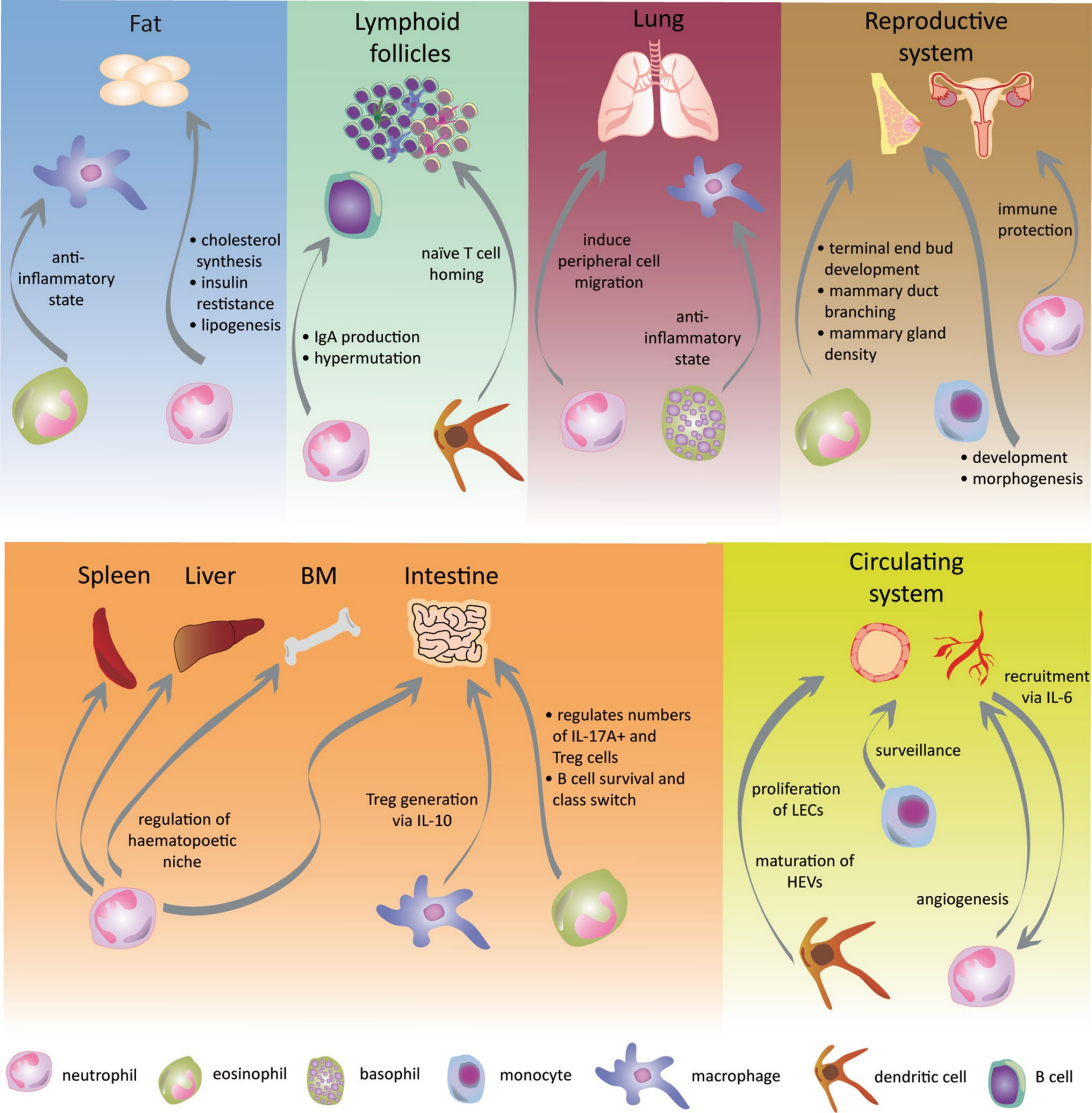
Heterogeneous functions of infiltrating myeloid cells in the steady-state. This figure summarizes the roles of granulocytes, monocytes and DC in different tissues during their infiltration in the steady-state. Neutrophils infiltrate most tissues following circadian rhythmicity not only as a mechanism of clearance but also contributing to organ performance. Eosinophils and basophils emerge as immunomodulators in several tissues such as the lung, adipose tissue, the female reproductive system and the intestine. Beyond their role in the adaptive immune response, DC regulate both lymphatic and blood endothelial cell proliferation through their migration to the lymph nodes. Similarly, in newly engrafted tissues, neutrophils can display pro-angiogenic effects

Myeloid cells, and in particular granulocytes, are recognized effectors of the immunomodulatory aspects during the steady-state, necessary to maintain tissue homeostasis (Fig. 1). Moreover, the infiltration of naïve tissues by granulocytes has been proved relevant for the function of multiple organs. The exact pathways by which particular tissue microenvironments determine the recruitment, migration and effects of granulocytes to perform homeostatic roles are questions that remain open in the field. We and others showed several mechanisms by which neutrophils infiltrate several tissues in the steady-state contributing to their normal function, beyond their immunomodulatory effects.

These novel roles of granulocytes in homeostasis raise interesting issues regarding their life cycle and migratory characteristics. The infiltration of naïve tissues by myeloid cells may follow specific patterns that differ from their migratory characteristics during inflammation. In fact, neutrophils show a probing migratory behavior in the steady-state, while their migration towards sites of injury and inflammation follow a directional mode [77]. However, how these migratory characteristics determine their later functions in the infiltrated tissue remains to be totally clarified.

An important outcome of this research focus will be the potential therapeutic approaches of modulating the infiltration of certain tissues by circulating myeloid cells to recover the normal function of damaged tissues. Future studies employing animal models of homeostasis and disease require the consideration of the growing knowledge on the heterogeneity of circulating myeloid cells and that some of these subsets might be predetermined to migrate selectively to different tissues. Single-cell sequencing technologies and mouse models of fate mapping are excellent tools to address these issues, as well as novel in vitro strategies to reprogram myeloid cells as potential cell-based therapeutic approaches.

## References

[1] Nourshargh S, Alon R (2014). Leukocyte migration into inflamed tissues. Immunity.

[2] Casanova-Acebes M, Nicolás-Ávila JA, Li JL (2018). Neutrophils instruct homeostatic and pathological states in naive tissues. J Exp Med.

[3] Reglero-Real N, Rolas L, Nourshargh S (2019). leukocyte trafficking: time to take time seriously. Immunity.

[4] Hadis U, Wahl B, Schulz O (2011). Intestinal tolerance requires gut homing and expansion of FoxP3+ regulatory T cells in the lamina propria. Immunity.

[5] Puga I, Cols M, Barra CM (2011). B cell-helper neutrophils stimulate the diversification and production of immunoglobulin in the marginal zone of the spleen. Nat Immunol.

[6] He W, Holtkamp S, Hergenhan SM (2018). Circadian expression of migratory factors establishes lineage-specific signatures that guide the homing of leukocyte subsets to tissues. Immunity.

[7] Semerad CL, Liu F, Gregory AD (2002). G-CSF is an essential regulator of neutrophil trafficking from the bone marrow to the blood. Immunity.

[8] Christopher MJ, Rao M, Liu F (2011). Expression of the G-CSF receptor in monocytic cells is sufficient to mediate hematopoietic progenitor mobilization by G-CSF in mice. J Exp Med.

[9] Bajrami B, Zhu H, Kwak H-J (2016). G-CSF maintains controlled neutrophil mobilization during acute inflammation by negatively regulating CXCR2 signaling. J Exp Med.

[10] Pillay J, den Braber I, Vrisekoop N (2010). In vivo labeling with 2H2O reveals a human neutrophil lifespan of 5.4 days. Blood.

[11] Furze RC, Rankin SM (2008). Neutrophil mobilization and clearance in the bone marrow. Immunology.

[12] Adrover JM, Nicolás-Ávila JA, Hidalgo A (2016). Aging: a temporal dimension for neutrophils. Trends Immunol.

[13] Casanova-Acebes M, Pitaval C, Weiss LA (2013). Rhythmic modulation of the hematopoietic niche through neutrophil clearance. Cell.

[14] Casanova-Acebes M, Pitaval C, Weiss LA (2013). XRhythmic modulation of the hematopoietic niche through neutrophil clearance. Cell.

[15] Scheiermann C, Kunisaki Y, Frenette PS (2013) Circadian control of the immune system. Nat Rev Immunol 10.1038/nri3386 PMC4090048 23391992

[16] Scheiermann C, Kunisaki Y, Lucas D (2012). Adrenergic nerves govern circadian leukocyte recruitment to tissues. Immunity.

[17] Wang J-X, Bair AM, King SL (2012). Ly6G ligation blocks recruitment of neutrophils via a β2-integrin–dependent mechanism. Blood.

[18] Zhang D, Chen G, Manwani D (2015). Neutrophil ageing is regulated by the microbiome. Nature.

[19] Casanova-Acebes M, Nicolás-Ávila JA, Yao Li JL (2018). Neutrophils instruct homeostatic and pathological states in naive tissues. J Exp Med.

[20] Stark MA, Huo Y, Burcin TL (2005). Phagocytosis of apoptotic neutrophils regulates granulopoiesis via IL-23 and IL-17. Immunity.

[21] Summers C, Rankin SM, Condliffe AM (2010). Neutrophil kinetics in health and disease. Trends Immunol.

[22] Gordy C, Pua H, Sempowski GD, He Y (2011). Regulation of steady-state neutrophil homeostasis by macrophages. Blood J.

[23] A-Gonzalez N, Quintana JA, García-Silva S (2017). Phagocytosis imprints heterogeneity in tissue-resident macrophages. J Exp Med.

[24] Geering B, Simon HU (2011). Peculiarities of cell death mechanisms in neutrophils. Cell Death Differ.

[25] A-Gonzalez N, Quintana JA, García-Silva S, et al (2017) Phagocytosis imprints heterogeneity in tissue-resident macrophages. J Exp Med 10.1084/jem.20161375 10.1084/jem.20161375 PMC5413334 28432199

[26] Lien DC, Wagner WW, Capen RL (1987). Physiological neutrophil sequestration in the lung: visual evidence for localization in capillaries. J Appl Physiol.

[27] Hogg JC, Walker BA (1995). Polymorphonuclear leucocyte traffic in lung inflammation. Thorax.

[28] Doyle NA, Bhagwan SD, Meek BB (1997). Neutrophil margination, sequestration, and emigration in the lungs of L-selectin-deficient mice. J Clin Invest.

[29] Devi S, Wang Y, Chew WK (2013). Neutrophil mobilization via plerixaformediated CXCR4 inhibition arises from lung demargination and blockade of neutrophil homing to the bone marrow. J Exp Med.

[30] Kreisel D, Nava RG, Li W (2010). In vivo two-photon imaging reveals monocyte-dependent neutrophil extravasation during pulmonary inflammation. Proc Natl Acad Sci.

[31] Cohen M, Giladi A, Gorki A-D (2018). Lung single-cell signaling interaction map reveals basophil role in macrophage imprinting. Cell.

[32] Chhiba KD, Hsu C-L, Berdnikovs S, Bryce PJ (2017). Transcriptional heterogeneity of mast cells and basophils upon activation. J Immunol.

[33] Schneider E, Petit-Bertron A-F, Bricard R (2009). IL-33 activates unprimed murine basophils directly in vitro and induces their in vivo expansion indirectly by promoting hematopoietic growth factor production. J Immunol.

[34] Sundd P, Pospieszalska MK, Ley K (2013). Neutrophil rolling at high shear: flattening, catch bond behavior, tethers and slings. Mol Immunol.

[35] Salvermoser M, Begandt D, Alon R, Walzog B (2018). Nuclear deformation during neutrophil migration at sites of inflammation. Front Immunol.

[36] Auffray C, Fogg D, Garfa M (2007). Monitoring of blood vessels and tissues by a population of monocytes with patrolling behavior. Science (80- ).

[37] Carlin LM, Stamatiades EG, Auffray C (2013). Nr4a1-dependent Ly6Clow monocytes monitor endothelial cells and orchestrate their disposal. Cell.

[38] Guilliams M, Mildner A, Yona S (2018). Review developmental and functional heterogeneity of monocytes. Immunity.

[39] Thomas G, Tacke R, Hedrick CC, Hanna RN (2015). Nonclassical patrolling monocyte function in the vasculature. Arterioscler Thromb Vasc Biol.

[40] Quintar A, McArdle S, Wolf D (2017). Endothelial protective monocyte patrolling in large arteries intensified by western diet and atherosclerosis. Circ Res.

[41] Merad M, Sathe P, Helft J (2013). The Dendritic cell lineage: ontogeny and function of dendritic cells and their subsets in the steady state and the inflamed setting. Annu Rev Immunol.

[42] Moussion C, Girard JP (2011). Dendritic cells control lymphocyte entry to lymph nodes through high endothelial venules. Nature.

[43] Webster B, Ekland EH, Agle LM (2006). Regulation of lymph node vascular growth by dendritic cells. J Exp Med.

[44] Wendland M, Willenzon S, Kocks J (2011). Lymph node T cell homeostasis relies on steady state homing of dendritic cells. Immunity.

[45] Braun A, Worbs T, Moschovakis GL (2011). Afferent lymph–derived T cells and DCs use different chemokine receptor CCR7–dependent routes for entry into the lymph node and intranodal migration. Nat Immunol.

[46] Kuravi SJ, McGettrick HM, Satchell SC (2014). Podocytes regulate neutrophil recruitment by glomerular endothelial cells via il-6–mediated crosstalk. J Immunol.

[47] Kolaczkowska E, Kubes P (2012). Angiogenic neutrophils: a novel subpopulation paradigm. Blood.

[48] Christoffersson G, Vågesjö E, Vandooren J (2012). VEGF-Arecruits a proangiogenic MMP-9-delivering neutrophil subset that induces angiogenesis in transplanted hypoxic tissue. Blood.

[49] Talukdar S, Oh DY, Bandyopadhyay G (2012). Neutrophils mediate insulin resistance in mice fed a high-fat diet through secreted elastase. Nat Med.

[50] Molofsky AB, Nussbaum JC, Liang H-E (2013). Innate lymphoid type 2 cells sustain visceral adipose tissue eosinophils and alternatively activated macrophages. J Exp Med.

[51] Wu D, Molofsky AB, Liang H-E (2011). Eosinophils sustain adipose alternatively activated macrophages associated with glucose homeostasis. Science (80- ).

[52] Chu DK, Jimenez-Saiz R, Verschoor CP (2014). Indigenous enteric eosinophils control DCs to initiate a primary Th2 immune response in vivo. J Exp Med.

[53] Chu VT, Beller A, Rausch S (2014). Eosinophils promote generation and maintenance of immunoglobulin-A-expressing plasma cells and contribute to gut immune homeostasis. Immunity.

[54] Matthews AN, Friend DS, Zimmermann N (1998). Eotaxin is required for the baseline level of tissue eosinophils. Proc Natl Acad Sci USA.

[55] Hogan SP, Mishra A, Brandt EB (2000). A critical role for eotaxin in experimental oral antigen-induced eosinophilic gastrointestinal allergy. Proc Natl Acad Sci USA.

[56] Jung Y, Wen T, Mingler MK (2015). IL-1β in eosinophil-mediated small intestinal homeostasis and IgA production. Mucosal Immunol.

[57] Sugawara R, Lee E-J, Jang MS (2016). Small intestinal eosinophils regulate Th17 cells by producing IL-1 receptor antagonist. J Exp Med.

[58] Chu VT, Fröhlich A, Steinhauser G (2011). Eosinophils are required for the maintenance of plasma cells in the bone marrow. Nat Immunol.

[59] Berek C (2016). Eosinophils: important players in humoral immunity. Clin Exp Immunol.

[60] Wira CR, Rodriguez-Garcia M, Patel MV (2015). The role of sex hormones in immune protection of the female reproductive tract. Nat Rev Immunol.

[61] Tessier DR, Raha S, Holloway AC (2015). Characterization of immune cells and cytokine localization in the rat utero-placental unit mid- to late gestation. J Reprod Immunol.

[62] Gouon-Evans V, Lin EY, Pollard JW (2002). Requirement of macrophages and eosinophils and their cytokines/chemokines for mammary gland development. Breast Cancer Res.

[63] Colbert DC, McGarry MP, O’neill K (2005). Decreased size and survival of weanling mice in litters of IL-5-/ -mice are a consequence of the IL-5 deficiency in nursing dams. Contemp Top Lab Anim Sci.

[64] Gouon-Evans V, Rothenberg ME, Pollard JW (2000). Postnatal mammary gland development requires macrophages and eosinophils. Development.

[65] Knudsen UB, Uldbjerg N, Rechberger T, Fredens K (1997). Eosinophils in human cervical ripening. Eur J Obstet Gynecol Reprod Biol.

[66] Gouon-Evans V, Pollard JW (2001). Eotaxin Is Required for eosinophil homing into the stroma of the pubertal and cycling uterus. Endocrinology.

[67] Tagliani E, Shi C, Nancy P (2011). Coordinate regulation of tissue macrophage and dendritic cell population dynamics by CSF-1. J Exp Med.

[68] Van Nguyen A, Pollard JW (2002). Colony stimulating factor-1 is required to recruit macrophages into the mammary gland to facilitate mammary ductal outgrowth. Dev Biol.

[69] Schwertfeger KL, Rosen JM, Cohen DA (2006). Mammary gland macrophages: pleiotropic functions in mammary development. J Mammary Gland Biol Neoplasia.

[70] Ingman WV, Wyckoff J, Gouon-Evans V (2006). Macrophages promote collagen fibrillogenesis around terminal end buds of the developing mammary gland. Dev Dyn.

[71] Ginhoux F, Guilliams M (2016). Tissue-resident macrophage ontogeny and homeostasis. Immunity.

[72] Bonnardel J, Guilliams M (2018). Developmental control of macrophage function. Curr Opin Immunol.

[73] Grainger JR, Wohlfert EA, Fuss IJ (2013). Inflammatory monocytes regulate pathologic responses to commensals during acute gastrointestinal infection. Nat Med.

[74] De Schepper S, Verheijden S, Aguilera-Lizarraga J (2018). Self-maintaining gut macrophages are essential for intestinal homeostasis. Cell.

[75] Munro DA, Wineberg Y, Tarnick J (2019). Macrophages restrict the nephrogenic field and promote endothelial connections during kidney development. Elife.

[76] Mass E, Ballesteros I, Farlik M (2016). Specification of tissue-resident macrophages during organogenesis. Science.

[77] Ng LG, Qin JS, Roediger B (2011). Visualizing the neutrophil response to sterile tissue injury in mouse dermis reveals a three-phase cascade of events. J Invest Dermatol.

